# Strangulation by Hair: An Uncommon Cause of a Common Accident in Children

**DOI:** 10.7759/cureus.87502

**Published:** 2025-07-08

**Authors:** Haya Alsarrawi, Abdelrahman Masri

**Affiliations:** 1 Pediatrics, University of Arkansas for Medical Sciences, Little Rock, USA; 2 Cardiology, University of Arkansas for Medical Sciences, Little Rock, USA

**Keywords:** accidental injury, child safety, hair strangulation, household hair, pediatric strangulation, suffocation hazard

## Abstract

Unintentional injuries are a leading cause of pediatric morbidity and mortality, with suffocation being a significant contributor. While common mechanisms include choking and entrapment, strangulation by household items such as human hair, known as hair-thread tourniquet syndrome (HTTS), is exceedingly rare and underreported. This report describes a rare case of a toddler who briefly lost consciousness after becoming entangled in a sibling’s long hair. Rapid parental response and emergency care led to a full recovery, but the incident underscores how everyday items can pose significant risks to young children, reinforcing the need for heightened awareness and proactive safety measures at home.

## Introduction

In the United States, there were approximately 1,040 deaths from accidental suffocation and strangulation in bed in 2022 [1]. In 2019, about 3,400 families experienced the loss of an infant due to sudden death, including accidental suffocation and strangulation in bed, sudden infant death syndrome (SIDS), which falls under the umbrella of unknown causes, and other unexplained deaths [2]. Clinical presentation of strangulation can vary widely depending on the mechanism and severity. The signs and symptoms may include cough, stridor, hoarseness, neck tenderness, petechiae, and subconjunctival hemorrhage. Notably, about 50% of strangulation victims may have no visible external signs of injury, and up to two-thirds may be asymptomatic initially [3]. In milder cases, a thorough physical examination is essential to avoid missed or delayed diagnoses [4].

Emergency care focuses on early recognition and securing the airway due to the high risk of progressive edema and airway obstruction. Early intubation, contingency planning, and appropriate airway equipment are critical [5]. Complications may include pulmonary edema and pneumonia [3], acute respiratory distress syndrome (ARDS), and increased intracranial pressure, particularly in patients with altered mental status [5]. Neurological symptoms may suggest vascular injury, such as carotid dissection, for which CT angiography (CTA) plays a key diagnostic role [5]. Children under the age of five are particularly vulnerable to airway compromise following strangulation because of their smaller laryngeal structures and more compliant soft tissues [6]. Even minimal swelling or hematoma formation can cause significant airway narrowing and respiratory distress [7].

Hair-thread tourniquet syndrome (HTTS) is a disorder where a strand of hair or thread tightly wraps around a body part, such as a finger, toe, or genitalia [8]. This report discusses a case of HTTS causing non-fatal strangulation, a very unusual presentation. Given the preventable nature of many strangulation injuries, prevention strategies are vital. Caregivers should be educated on common hazards as a routine part of anticipatory guidance by pediatric healthcare providers.

This article was previously presented as a poster at the 2025 Southern Regional Meeting on February 15, 2025.

## Case presentation

**Figure 1:**
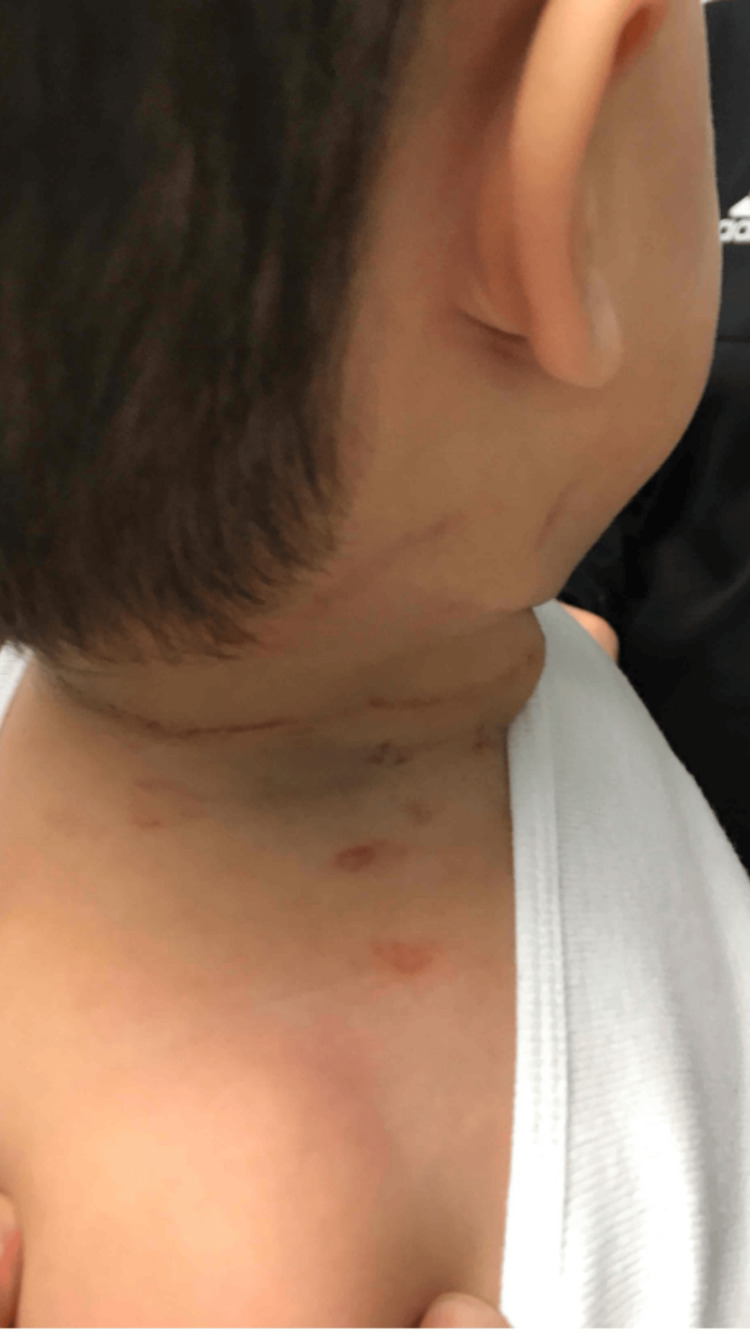
Excoriation on the patient's neck A linear excoriation with erythematous and bluish discoloration was observed around the neck, attributed to both hair strangulation and parental attempts to remove the hair using their fingernails

A 15-month-old male with no significant medical history was brought to the ED after an accidental strangulation incident at home. The child’s 14-year-old sister, who kept her knee-length hair loose, had been lying on the floor when the child had crawled into her hair. Upon her movement, the hair had tightened around his neck, causing immediate cyanosis. The parents had attempted manually to release the hair, causing excoriation (Figure 1), and then they had used scissors to cut the hair, successfully freeing the child within one minute.

**Figure 2:**
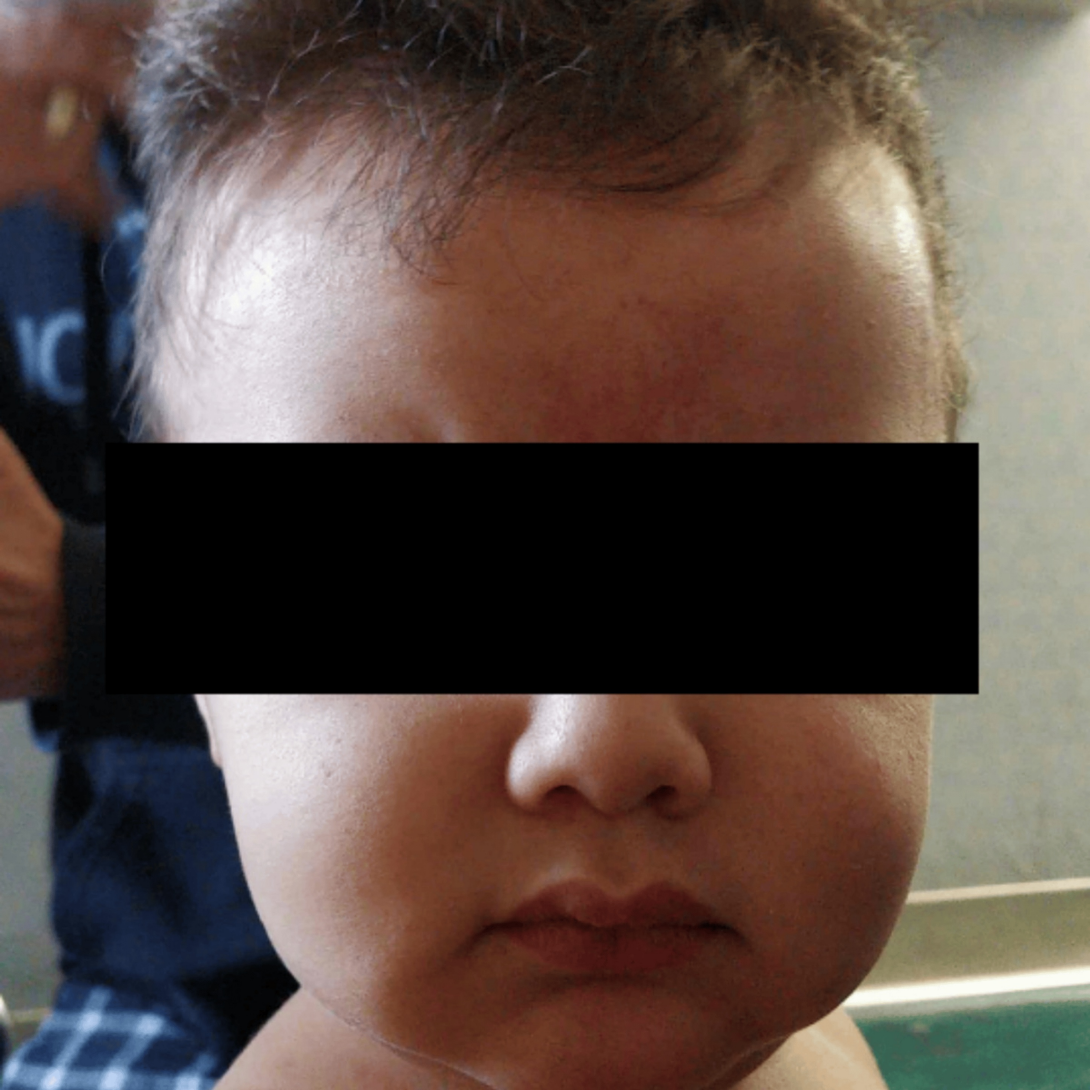
Facial petechiae The image shows multiple pinpoint, non-blanching petechiae across the forehead and periorbital regions, consistent with increased venous pressure from transient neck compression due to hair strangulation

Upon regaining consciousness, the child had been noted to be limp and unresponsive for approximately 30 seconds. The parents had performed chest compressions and mouth-to-mouth resuscitation during this period. In the ED, the child exhibited inspiratory stridor, facial petechiae (Figure 2), and subconjunctival hemorrhage. Trauma surgery was consulted, a cervical collar was applied, and imaging studies, including a CTA of the head and neck, were performed, revealing no vascular injuries.

The patient was given one dose of dexamethasone for stridor and observed overnight. A dermatology consult was not obtained due to the quick resolution of skin findings. He was discharged the following day in stable condition. A child abuse consultation confirmed the incident to be accidental, and the family was advised on safety measures, including tying the long hair back to prevent similar incidents. The patient was observed to have normal development during his subsequent pediatric visit.

## Discussion

We presented a case of a 15-month-old male who experienced accidental strangulation due to his sister's loose hair. This aligns with existing literature highlighting HTTS as a rare but serious condition in young children. HTTS typically involves a strand of hair or thread becoming tightly wrapped around an appendage, leading to vascular or tissue damage [8]. A literature review of HTTS cases emphasizes the importance of early recognition and intervention to prevent complications such as tissue necrosis or airway obstruction. While most cases involve digits or genitalia, neck involvement is less common but has been documented [9,10].

Our patient exhibited inspiratory stridor, facial petechiae, and subconjunctival hemorrhage upon presentation, which are consistent with findings in previous reports. For instance, a 13-month-old male presented with similar symptoms after being found entangled in his mother's hair during co-sleeping [11]. These manifestations are indicative of venous congestion and increased intracranial pressure resulting from the constriction of the neck. In our case, imaging studies, including a CTA of the head and neck, were performed to rule out vascular injuries. This approach is consistent with current practices, as detailed evaluations are essential to differentiate accidental strangulation from non-accidental trauma [12]. Previous reports have similarly involved comprehensive diagnostic workups to ensure accurate diagnosis and appropriate management [9,10,11]. The child was treated supportively and discharged in stable condition, consistent with recommended HTTS management focusing on prompt removal of the constricting material and observation [13].

Based on current knowledge, all reported cases of hair strangulation have been associated with co-sleeping, particularly the one involving the neck [11]. However, our case demonstrates that long hair can pose a potential hazard to toddlers even in positions other than co-sleeping. Rare cases of hair tourniquets used as a form of child abuse have been described, particularly in the context of a caregiver deliberately applying a genital tourniquet to prevent nocturnal enuresis. Although such instances are exceedingly rare, unintentional hair tourniquets are more common and typically accidental [14]. Nonetheless, it is essential to always rule out abuse in these cases, with a strong emphasis on performing a detailed physical examination [15].

## Conclusions

This report highlights a rare presentation of HTTS involving neck entanglement during normal daytime activity, expanding our knowledge about common organs involved and the risk of strangulation outside the context of co-sleeping. There may be value in pediatric anticipatory guidance emphasizing the risk posed by unsecured long hair and encouraging caregivers to take preventive measures, such as tying the hair back, to reduce injury risk.
